# The Impact of Purinergic System Enzymes on Noncommunicable, Neurological, and Degenerative Diseases

**DOI:** 10.1155/2018/4892473

**Published:** 2018-08-12

**Authors:** Margarete Dulce Bagatini, Alessandra Antunes dos Santos, Andréia Machado Cardoso, Aline Mânica, Cristina Ruedell Reschke, Fabiano Barbosa Carvalho

**Affiliations:** ^1^Coordenação Acadêmica, Universidade Federal da Fronteira Sul, Campus Chapecó, Chapecó, SC, Brazil; ^2^Programa de Pós-graduação em Ciências Biológicas-Bioquímica Toxicológica, Universidade Federal de Santa Maria, Santa Maria, RS, Brazil; ^3^Department of Molecular Pharmacology, Albert Einstein College of Medicine, Bronx, NY, USA; ^4^Department of Physiology and Medical Physics, Royal College of Surgeons in Ireland, Dublin 2, Ireland; ^5^Laboratório de Pesquisa em Patologia, Universidade Federal de Ciências da Saúde de Porto Alegre, Porto Alegre, RS, Brazil

## Abstract

Evidences show that purinergic signaling is involved in processes associated with health and disease, including noncommunicable, neurological, and degenerative diseases. These diseases strike from children to elderly and are generally characterized by progressive deterioration of cells, eventually leading to tissue or organ degeneration. These pathological conditions can be associated with disturbance in the signaling mediated by nucleotides and nucleosides of adenine, in expression or activity of extracellular ectonucleotidases and in activation of P2X and P2Y receptors. Among the best known of these diseases are atherosclerosis, hypertension, cancer, epilepsy, Alzheimer's disease (AD), Parkinson's disease (PD), and multiple sclerosis (MS). The currently available treatments present limited effectiveness and are mostly palliative. This review aims to present the role of purinergic signaling highlighting the ectonucleotidases E-NTPDase, E-NPP, E-5′-nucleotidase, and adenosine deaminase in noncommunicable, neurological, and degenerative diseases associated with the cardiovascular and central nervous systems and cancer. In conclusion, changes in the activity of ectonucleotidases were verified in all reviewed diseases. Although the role of ectonucleotidases still remains to be further investigated, evidences reviewed here can contribute to a better understanding of the molecular mechanisms of highly complex diseases, which majorly impact on patients' quality of life.

## 1. Introduction

Noncommunicable, neurological, and degenerative diseases are characterized by cell loss, ultimately leading to deterioration in quality or function of tissues or organs and possible failure of vital organs [1]. Although the etiology and pathogenesis of these diseases remain unclear, recent advances indicate that the processes of organ deterioration share common core features, including cell injury and dysfunction that contribute to functional and morphological impairment of cells. Despite considerable progress in understanding the molecular mechanisms of these diseases, current therapeutic options are limited, and no effective pharmacological treatment has emerged to date. Elucidation of common and unique mechanisms responsible for the deterioration present in these pathologies may facilitate the identification and development of effective targets and therapies [2]. Furthermore, the search for specific (bio) markers for each human condition—physiological and pathological—is becoming critical.

Elements of the purinergic signaling system are involved in many processes in health and disease conditions [3]. Therefore, a complete understanding of purinergic system could potentially unveil possible markers or relevant pathways for pathological processes, mainly related to human degeneration. Briefly, the purinergic system consists of three main components: (i) the extracellular nucleotides and nucleosides, which mediate signaling; (ii) the receptors through which these nucleotides and nucleosides exert their effects; (iii) and the ectoenzymes, responsible for the control of extracellular levels of these molecules [4]. The control of the levels of extracellular nucleotides adenine and adenosine and the consequent signaling by purinergic receptors induced by them is critical in maintaining the physiological processes [5]. This control is performed by ectonucleotidases, which are enzymes anchored to the cell surface or located in the interstitial medium (soluble form) [6].

## 2. Purinergic System

Purines' extracellular role was first demonstrated in 1929 by Drury and Szent-Györgyi [7], which described its actions in mammary hearts [8–10]. Although, only in 1970, Burnstock proposed the term “purinergic” and presented his hypothesis about ATP as an independent neurotransmitter released from nonadrenergic noncholinergic neurons in the intestines, bladder, and gut [11, 12]. Two years later, Burnstock described adenosine triphosphate (ATP) as an extracellular signaling molecule and its effects [13]. However, the purinergic system and ATP had an arduous path to be accepted by the scientific community. Only in 2006, ATP was finally recognized as a cotransmitter in both the peripheral and central nervous systems (CNS) [9, 10, 14], and the purinergic signaling was recognized as a system involved in many nonneuronal and neuronal mechanisms [12].

ATP is the most versatile nucleotide and the primary energy source for cellular functions. Hundreds of reactions in the cell, from metabolic transformations to signaling events, are coupled to the hydrolysis of ATP [15]. Intracellularly, ATP is stored at very high levels (from 5 to 10 mmol/l), which can quickly be degraded by ubiquitous extracellular nucleotidases after connecting to specific receptors under physiological conditions. In fact, extracellular ATP has an extremely short half-life before it is degraded to adenosine—milliseconds to seconds. This rapid breakdown results in the activation of a multiplicity of receptor subtypes, which can mediate physiological processes such as proliferation, differentiation, migration, and cell death [16]. On the other hand, the excess of ATP in the brain extracellular space can induce neurotoxicity [17].

ATP stores energy by losing a phosphate group and forming ADP. It has been shown that the ADP molecule can have an important role in platelet aggregation (platelet granules contain high concentrations of ADP), blood vessel tone, cardioprotection, and vascular wall integrity [18].

The critical functions of ATP and its subsequent hydrolysis are initiated upon binding to purinergic receptors, such as P2 nucleotide and P1 adenosine receptors [19]. Abbracchio and Burnstock divided P2 receptors into two families: P2X family of ligand-gated ion channel receptors and the P2Y family of G protein-coupled receptors, based on their molecular structure, induced mechanism of action, and the sequence analysis of cloned P2 receptors [20].

Currently, thirteen human P2X receptor subtypes can be distinguished: 6 homomeric (P2X1, P2X2, P2X3, P2X4, P2X5, and P2X7) and 7 heteromeric (P2X1/2, P2X1/4, P2X1/5, P2X2/3, P2X2/6, P2X4/6 [21], and P2X4/7) [22]. P2X receptors are nonselective ligand-gated ion channels that mediate sodium influx, potassium efflux, and at some extent calcium influx, leading to cell membrane depolarization [23]. The P2X2/3 receptors are located in the nodose ganglia [24], P2X4/6/7 in the CNS [25, 26], P2X1/5 in the blood vessels, and P2X2/6 receptors are mainly located in the brain stem [24, 27, 28]. P2X receptors have important functions in the central and peripheral nervous systems, such as slow neuromodulatory function, rapid synaptic transmission, neurotransmitter release, and the generation of pain signals. Furthermore, these receptors have pathophysiological role in injury, inflammation, anxiety, dementia, epilepsy [26], and neurodegenerative disorders such as Alzheimer's and Huntington's diseases [29–32].

P2Y receptors are G protein-coupled receptors, virtually present in all cells, and mainly activated by adenine and uridine nucleotides. There are eight subtypes of P2Y receptors (P2Y1, P2Y2, P2Y4, P2Y6, P2Y11, P2Y12, P2Y13, and P2Y14), which are ubiquitously expressed in body, including the CNS [32]. They activate intracellular signaling cascades to regulate a variety of cellular and peripheral pathophysiological processes, including inflammation, ischemia, and pain [32, 33]. In the brain, P2Y receptors exert important roles in neurotransmission, glia cell communication, and neurogenesis due to their localization on neurons, oligodendrocytes, microglia, and astrocytes [13, 34–36].

After release and binding to specific receptors, ATP and other nucleotides undergo rapid enzymatic degradation by ectonucleotidases [4]. Ectonucleotidase families include the ectonucleoside triphosphate diphosphohydrolases (E-NTPDase/CD39/NTPDase 1), ectonucleotide pyrophosphatase/phosphodiesterases (E-NPP), and ecto-5′-nucleotidase (E-5′-nucleotidase/CD73). E-NTPDases and E-NPPs hydrolyze ATP and ADP to adenosine monophosphate that is further hydrolyzed by E-5′-nucleotidase to adenosine [6, 37, 38].

Since 1929, adenosine has been recognized as a biologically significant molecule, responsible for regulating multiple systems including cardiac conduction, arterial pressure, and intestinal motility [7]. Adenosine is produced both intracellularly and extracellularly through enzymatic degradation of adenine nucleotides [39, 40]. The maintenance of its levels in the extracellular fluids is the result of the balance between its production and consumption [19]. Indeed, adenosine synthesis is controlled by ectonucleotidases located on the cytoplasmatic membrane, and its concentrations vary according to physiological and pathological stimuli such as hypoxia and inflammation [41, 42]. Extracellular adenosine is involved in many cytoprotective functions of the body, including conditioning the heart against ischemia, counteracting the damaging effects of excitotoxicity and seizure activity in the brain, and suppressing excessive immune and inflammatory responses. Finally, extracellular adenosine can be deaminated to inosine by adenosine deaminase (ADA) [43].

Critically, the understanding of the physiological and pharmacological roles of adenosine was greatly facilitated by localizing the different P1 receptor subtypes in target tissues and identifying its four subtypes—A1, A2A, A2B, and A3 receptors [44–46]. These are G protein-coupled receptors, which present inhibitory action upon adenosine binding [20]. P1 receptors are widely located in the brain and in peripheral tissues, such as the heart, kidney, and adrenals of different species [45, 46].

**Figure 1:**
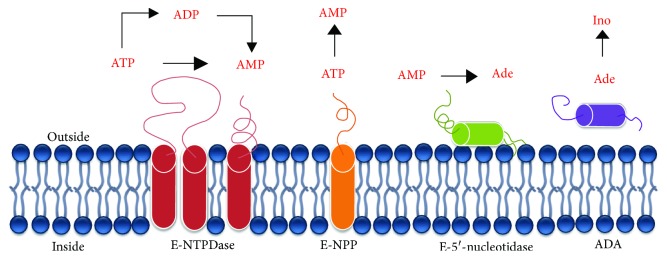
Cell membrane-anchored ectonucleotidases and their respective hydrolysis reactions.

After the binding to receptors, the nucleotides and nucleosides are rapidly degraded by specific hydrolase enzymes, which occurs via different ways [47]. The E-NTPDase catalyzes ATP and ADP hydrolyses, which culminates into AMP formation. E-5′-nucleotidase is responsible for AMP degradation leading to adenosine formation. Alkaline phosphatase removes inorganic phosphate (Pi) of a broad range of substrates, including nucleotides (ATP, ADP, and AMP) producing adenosine (Figure 1) [48, 49]. The producing adenosine is converted into inosine by adenosine deaminase (ADA) [38].

The E-NTPDase family is very efficient in controlling the bioavailability of ATP [49]. This family consists of eight members, E-NTPDases 1–8, which differ in substrate specificity, tissue distribution, and cellular localization [50]. Members 1, 2, 3, and 8 are the principal enzymes responsible for the hydrolysis of tri- and diphosphate nucleotides on the cell surface under physiological conditions. E-NTPDases 4, 5, 6, and 7 are associated with intracellular organelles [50]. These ectoenzymes are anchored to the plasma membrane via hydrophobic domains with the active site facing the extracellular medium [5, 38, 48].

The E-5′-nucleotidase family is comprised of seven isolated and characterized isoforms with different nomenclature, depending on the subcellular localization. Five isoforms are cytosolic, one is located in the mitochondrial matrix, and one is related to the outer plasma membrane [47]. This enzyme generates extracellular adenosine from AMP, as previously mentioned, and its activity is the rate-limiting step of adenosine formation from adenine nucleotides in most tissues [49, 51].

The E-NPPs are transmembrane glycoprotein type II enzymes, which are able to catalyze many different reactions: 3′-5′-cyclic adenosine monophosphate (5′-cAMP) to AMP; ATP to AMP and inorganic diphosphate (PPi); AMP to ADP and inorganic phosphate (Pi); nicotinamide adenine dinucleotide oxidized (NAD^+^) to AMP and mononucleotide nicotinamide [6]. Although this family of enzymes is composed by 7 members, only E-NPPs 1, 2, and 3 are able to hydrolyze nucleotides [5].

ADA activity has been found altered in various pathological conditions including acquired immunodeficiency syndrome (AIDS), anemia, lymphomas, tuberculosis, and leukemia [47]. In humans, different ADA isoforms have been identified: adenosine deaminase-1 (ADA-1), adenosine deaminase-2 (ADA-2), and adenosine deaminase-3 (ADA-3) [52]. ADA-1 plays a metabolic role not only as a key cytosolic enzyme in the purine pathway but also as an ectoenzyme by regulating extracellular adenosine levels. In contrast, ADA-2 is specifically designed to act in the extracellular environment according to its presence in the serum [53]. Thus, ADA can be considered as a multifunctional protein, playing several roles and importantly regulating biological systems, which consequently impacts on human health [53].

## 3. Noncommunicable, Neurological, and Degenerative Diseases

According to the World Health Organization (WHO), chronic disorders account for 38 million deaths each year, more than 80% of all deaths worldwide. Cardiovascular diseases cause most of the deaths (17.5 million per annum), followed by cancers (8.2 million), respiratory diseases (4 million), and diabetes (1.5 million) [54, 55]. In the last two decades, this profile of morbidity and mortality causes around the world has significantly been changed, and, increasingly, chronic neurological and degenerative diseases are becoming more prominent [54].

Among the better-known chronic diseases are the noncommunicable, such as atherosclerosis, hypertension, diabetes, and cancer; the neurological, such as epilepsy; and the neurodegenerative, such as Alzheimer's disease (AD), Parkinson's disease (PD), and multiple sclerosis (MS). The study and control of these diseases are exceedingly difficult due to their multiplicity and diversity, for instance, the interconnection network of risk and protective factors, diverse onsets followed by multistep pathogenesis, and, in some cases, multifocal localization [56]. A common feature among many of the neurological, neurodegenerative, and several aspects of cardiovascular diseases may be a component of degeneration. It means that at some stage occurs loss of cells, subcellular function, or tissue elements or function [57]. In fact, these diseases may also share other pathological features or events, such as evidence of membrane damage, oxidative stress, mitochondrial dysfunction, and upregulation of autophagy [58].

Once purinergic signaling is involved in virtually all body functions, the roles of purines might be altered in pathological states in different body systems [59]. Indeed, purinergic mechanisms and specific receptor subtypes have been shown to be involved in several pathologies including brain trauma and ischemia, neurodegenerative diseases involving neuroimmune and neuroinflammatory reactions [60], diabetes [61], vascular diseases including atherosclerosis [62, 63], and hypertension [9, 10]. Importantly, studies have shown the potential of purinergic mechanisms as therapeutic targets for the treatment of neurological [36] and degenerative disorders [9, 64, 65]. Moreover, the currently used strategies in searching for novel purinergic targeting drugs include the development of (i) selective agonist and antagonist ligands for the P2X and P2Y receptors, (ii) inhibitors of extracellular catabolism of purines, and (iii) modulators of nucleotide and nucleoside transport [66]. Adenosine signaling manipulation may also have therapeutic potential in neurodegenerative diseases such as AD, PD, and Huntington's diseases and in neurological and psychiatric disorders such as epilepsy [67], schizophrenia, and autism [68].

Taking that into account, the next section provides an overview of recent findings regarding the role of purinergic system enzymes in the pathophysiology of noncommunicable, neurological, and degenerative diseases. Considerable attention has been directed towards diseases related to the cardiovascular system (atherosclerosis and hypertension), cancer, and central nervous system (epilepsy, Alzheimer's disease, Parkinson's disease, and multiple sclerosis).

### 3.1. Atherosclerosis

Atherosclerosis is popularly defined as an artery wall thickening triggered by accumulation of foam cells and proliferation of intimal smooth muscle cell, which results in a fibrofatty plaque. Atherosclerotic disease still is the worldwide leading cause of cardiovascular complications and death [69]. The drivers for atherosclerotic plaque development include fluid shear forces, lipid milieu, cells of the vascular wall, and cells recruited from the circulation. However, one of the central events that results in the atherosclerosis development is the disturbance in blood flow in human arteries. In addition, some cells, such as endothelial cells, platelets, monocytes, and T cells, can be involved and/or activated in response to changes in blood flow [70].

Arteries presenting constant flow are further resistant to plaque formation when compared to arteries presenting disturbance in blood flow. Taking this into account, it is important to point out that the aspects that control the blood flow, such as genes, microRNAs, and epigenomic processes, initiate responses that can result in activated or quiescent endothelial phenotypes and, in turn, susceptibility to or protection from atherosclerosis [70–72]. Corroborating with this, Nam and collaborators demonstrated that disturbed flow directly causes atherosclerosis within 14 days, under hyperlipidemic conditions, after partial carotid ligation surgery [71].

It is well stablished that E-NTPDase 1 is the major ectonucleotidase expressed in blood vessels. Several studies have been carried out in order to elucidate the role of this enzyme in controlling the blood flow, atherosclerosis development, and inflammation related to vascular disorders [70, 73] (Table 1). It has been widely shown that E-NTPDase 1 expressed in both endothelial and smooth muscle cells, along with autonomic nervous system, can control the contraction of arteries, which directly influences the regulation of blood circulation [49, 74–77]. Moreover, E-NTPDase 1 plays a critical role limiting the activation of some P2 receptors, thus, preventing its desensitization in endothelial and smooth muscle cells, consequently, preventing excessive artery contraction [76–79]. This fact can be explained because ATP, released from sympathetic nerves, along with norepinephrine (NE) and neuropeptide Y, can activate P2X receptors at postjunctional membranes evoking vascular smooth muscle contraction, which generally potentiate the effects of NE at *α*1-adrenoceptors [51, 80]. Accordingly, Cardoso et al. have shown that E-NTPDase 1 expression and activity have the ability to control the effects of sympathetic nervous system in the vasculature [81].

Regarding to the vascular injury related to atherogenesis, it has been shown that E-NTPDase 1 naturally results in protection from the action of nucleotides released from the injured cell in vasculature or activated platelets by eliminating these prothrombotic and proinflammatory stimuli, that is, by metabolizing extracellular ATP and ADP. In a recent work, Kanthi et al. have evaluated the role of E-NTPDase 1 in the development of atherogenesis, in an apolipoprotein E-deletion mouse model of atherosclerosis (ApoE-deficient) [70]. ApoE-deficient group, fed with a high-fat diet, showed evidently augmented and plaque formation along with platelet activation that was verified by some circulating markers. In this work, the authors also showed that in regions of stable flow, E-NTPDase 1 was markedly present in ApoE-deficient mice. In contrast, E-NTPDase 1 was reduced in atheroprone as subject to disturbed flow. Moreover, in this same work, it has been demonstrated that the deregulated flow triggered by a partial carotid artery ligation quickly suppressed endothelial E-NTPDase 1 expression. Furthermore, unidirectional laminar shear stress induced atheroprotective E-NTPDase 1 expression in human endothelial cells. In these conditions, the vascular transcription factor involved in the E-NTPDase 1 is the Krüppel-like factor 2 (KLF2), which can bind near the transcriptional start site of E-NTPDase 1.

Mercier and collaborators have shown that ApoE knockout mice displayed decreased E-NTPDase 1 expression and activity in the thoracic aorta, which correlates with reduced vascular reactivity and presence of atherosclerotic plaques in aortic roots and arches. The authors have speculated that the accumulation of ATP and ADP in ApoE knockout mice may cause desensitization of P2 receptors, which in turn may contribute to decreased blood flow and may predispose an individual to atheroma formation [82]. In a carotid artery wire injury model, E-NTPDase 1 knockout mice showed decreased migration of vascular smooth muscle cells and reduced neointimal formation, indicating that CD39 contributes to a harmful neointima formation [83]. Taken together, these data establish that the key regulator of atherosclesosis development driven by shear stress is the E-NTPDase 1 enzyme. Kanthi et al. have unveiled what is now believed to be a previously unrecognized role for E-NTPDase 1 as an endogenous regulator of endovascular purine levels, serving as a modulator of crucial cellular drivers of atherogenesis [70]. Emphasizing the role of E-NTPDase 1 in providing anti-inflammatory and antithrombotic mediator adenosine, it has been shown that therapeutic strategies targeting CD39 offer promising opportunities in the management of vascular thromboinflammatory diseases [73].

CD73 also has been reported playing a key role in the atherogenic process by driving purinergic signaling [84, 85]. In a work developed by Buchheiser et al., it was demonstrated that the inactivation of CD73 promotes atherogenesis in apolipoprotein E-deficient mice [86]. The ablation of CD73 in ApoE knockout animals triggered an augmentation in atheroma formation, which was probably generated by the reduced inhibitory control exerted by adenosine on immune cells, which are dynamically related to the atherogenic process [86]. The role of CD73 in protection against atherosclerosis was also observed after injury of carotid arteries. This condition results in augmented expression of vascular cell adhesion molecule- (VCAM-) 1 and increased nuclear factor-kappa B activity, which triggers the formation of neointimal plaque and continuous infiltration of macrophage in CD73 knockout animals when compared to control groups [87].

Jalkanen and collaborators have evaluated 226 patients with stable peripheral artery disease admitted for nonurgent invasive imaging and treatment. They verified that the progression of atherosclerosis is associated with low E-NTPDase 1 activity and high CD73 [88]. Same authors also found high levels of ATP and ADP in the plasma of those patients, suggesting that low E-NTPDase 1 activity is associated with ATP- and ADP-induced platelet aggregation and trombus formation [88].

Since adenosine is the main molecule that presents antithrombotic effects, ADA also has a key role in atherogenesis process. This enzyme has been suggested as an inflammatory marker [89], and its high expression and activity were associated with the risk of atherosclerosis development [89, 90]. Kalvegren et al. have verified changes in metabolism of extracellular nucleotides in the atherosclerotic vessel wall from aortoiliac bifurcation of apoliprotein E- and LDL-deficient animals (ApoE/LDLr (−/−)). They observed that the ADA activity was decreased as well as the levels of adenosine in ApoE/LDLr (−/−) mice when compared to littermate control, reinforcing the idea that low ADA activity is a predictor of atherogenesis. Another mechanism that can contribute to the development of atherosclerosis is that high levels of ADA can increase the release of reactive oxygen species from neutrophils, through a downregulation of the inhibitory adenosine/cAMP system and an enhanced activation of A1 receptors. This sustained neutrophil activation could also contribute to inflammatory disorders and atherogenesis [91].

Genetic studies have been performed regarding the relationship between ADA and atherosclerosis, and two main points can be highlighted: (1) single-nucleotide polymorphisms of the RNA-specific gene of ADA are associated with metabolic disorders in general and atherosclerosis [92] and (2) high ADA gene expression is related to the augmentation of cathepsin S mRNA, which encodes a cysteine protease associated with angiogenesis and atherosclerosis [93].

Taking the above information into account, it is possible to conclude that a high expression and activity of E-NTPDase 1 and CD73, in order to generate adenosine, and a low expression and activity of ADA, in order to keep the levels and let the adenosine molecule exerts its antithrombotic effects, may exert a positive effect in preventing atherosclerosis.

### 3.2. Hypertension

Hypertension is a highly prevalent disease, which is estimated to affect 26% of the worldwide adult population [94, 95]. Hypertension is defined by a systolic blood pressure (BP) of 140 mmHg or higher, a diastolic BP of 90 mmHg or higher, or currently using BP-lowering drugs [95]. The disease represents a major risk factor for the development of kidney failure, coronary events, cerebrum-vascular disease, heart failure, and peripheral vascular disease [94, 95]. Hypertension is the leading cause of morbidity and mortality worldwide while cigarette-smoking is the major preventable cause of death [96]. Nowadays, most patients require more than one drug to achieve BP target, and monotherapy would only be sufficient in about 20–30% of patients [95]. This fact highlights the unmet need to unveil other mechanisms that can be associated with hypertension development and maintenance.

Among the events related to hypertension, we can cite platelet activation and aggregation, vasoconstriction, and low-grade inflammatory status. It is well known that the purinergic system enzymes are tightly involved regulating nucleotides that can trigger or protect against platelet aggregation. Moreover, the control of extracellular circulating nucleotides by ectonucleotidases is related to both anti- and proinflammatory status [49]. Recently, Fabbiano and collaborators have demonstrated that regulatory T (TReg) cell-expressing E-NTPDase 1 require an immunosuppression-independent mechanism to counteract renal and possibly cardiac damage during angiotensin II- (AngII-) dependent hypertension [97]. Tissue-resident neutrophils suffer apoptosis by ATP hydrolysis of E-NTPDase 1. The same group also stated that genetic alterations in the number of TReg and TH cells has an influence on tissue-resident neutrophil number, cardiomyocyte hypertrophy, cardiorenal fibrosis, and elevation of arterial pressure during AngII-induced hypertension [97]. Thus, TReg cell-expressing E-NTPDase 1 can protect against hypertension-driven fibrosis in tissue.

In pulmonary arterial hypertension (PAH), two independent studies have been performed with different outcomes. Using human samples, *in vitro* strategies, and a rat model, Helenius et al. have demonstrated that the suppression of E-NTPDase 1 is linked to the pathogenesis of PAH [98]. Furthermore, they stated that the accumulation of extracellular ATP and ADP is strictly linked to vascular dysfunction and remodeling in PAH and can modulate the disease course in multiple levels. On the other hand, Visovatti et al. have observed that microparticles from platelets and endothelial cells of patients with PAH display increased E-NTPDase 1 activity and expression [99]. Therefore, more studies are crucial to elucidate whether high or low levels of E-NTPDase 1 can be related to PAH development.

Taking advantage of a preeclampsia mouse model, McRae et al. have evaluated the impact of E-NTPDase 1 overexpression. In this study, the authors injected Th1-polarized cells on pregnancy days 10 and 12 of wild-type and E-NTPDase 1 transgenic mice and measured the systolic blood pressure (SBP) until pregnancy day 15, when mice were sacrificed. Following transfer of Th1-polarized cells, SBP of E-NTPDase 1 transgenic mice remained unchanged, without evidence of renal lesions, while an increase was observed in pregnant wild-type mice. Thus, the authors have concluded that E-NTPDase 1 overexpression can be protective in a mouse model of preeclampsia [100].

Sympathetic system has a crucial link with hypertension development by controlling vascular tonus. E-NTPDase 2 is associated with the adventitia of muscularized vessels, microvascular pericytes, and other cell populations in the subendocardial space in the heart [75]. Later, Rücker et al. have shown that E-NTPDase 2 is the most expressed ectonucleotidase in synaptosomes prepared from rat heart left ventricles, indicating that this enzyme may be essential for modulating ATP and NE responses on heart fibers [101]. At the same time, the presence of E-NTPDase 2 in the vascular murine adventitial cells [75] also suggests that this enzyme plays a role in hydrolyzing the ATP released from sympathetic nerves and, thus, can help to control vascular tone and hypertension development.

E-5′-nucleotidase is also highly expressed in the left ventricle, and its activity seems to be important for the control of the nucleotide/nucleoside ratio in the vicinity of nerve endings in the heart [101]. Moreover, in human coronary arteries, sympathetic nerves are one of the sources of adenine nucleotides and coronary vasodilation was found to be associated with the endothelial expression of E-5′-nucleotidase. Inhibition of E-5′-nucleotidase and the usage of P1 antagonists have been associated with a marked reduction of the relaxation of coronary arteries [102]. The same occurs in mesenteric arteries [103] and other vascular beds [51]. Data from Sousa and colleagues have revealed that the increase in the sympathetic tonus in spontaneously hypertensive rats can be associated to a higher NE/ATP release ratio from sympathetic nerves and to deficits in the endogenous inhibitory tonus mediated by prejunctional adenosine A1 receptors [103]. This fact reinforces the important role of E-NTPDase 1, E-NTPDase 2, and CD73 in producing adenosine and preventing hypertension development caused by augmented sympathetic tonus. Indeed, adenosine produced by E-5′-nucleotidase activates P1 receptors, which can cause hyperpolarization and relaxation of the underlying vascular smooth muscle cells [102].

Increased E-NTPDase and CD73 activities have been observed in both animal models of hypertension, in human studies, and in platelets and lymphocytes [81, 104–107]. These specific platelet and lymphocyte responses can be understood as a mechanism to ameliorate hypertension through the elevation of adenosine levels, by combined actions of E-NTPDase and CD73.

Taken together, these results indicate that E-NTPDase 1 and CD73 expressed in lymphocytes, TReg cells, endothelial cells, and platelets constitute a protective barrier against hypertension-driven tissue fibrosis. In addition, the results suggest new therapeutic avenues to prevent hypertension and hypertension-linked pathologies.

Changes in ADA expression and activity are also related to hypertension development and maintenance. Human studies have shown increase in ADA activity and expression in response to hypertension and hypertension-associated pathologies, such as metabolic syndrome [108]. A genetic study with hypertensive patients showed that a common polymorphism (C34T) of the ADA gene (isoform 1) is strongly correlated with essential hypertension [109]. ADA activity is also increased in both platelets and lymphocytes in an animal model of hypertension induced by L-NAME administration [81, 104–106].

Moreover, Tofovic et al. have suggested that the inhibition of ADA may provide beneficial effects in old hypertensive animals, and an inhibitor of this enzyme could be designed and used to offer cardiovascular protection in hypertension [110]. Franco and colleagues have analyzed the activity of nucleotidases and ADA in cytosolic and membrane fractions of renal tissue, in an angiotensin-II model of hypertension [111]. They observed a decrease in the membrane ADA activity and expression, in AngII-treated rats. Furthermore, despite the adenosine elevation, A1 and A2B receptor protein expression did not change; in contrast, a downregulation was observed in A2A receptors and an upregulation in A3 receptor levels. A similar pattern was found in the cortex and in the medulla—the expression of A3 receptor decreased in both segments. These results suggest that the elevation of renal tissue and interstitial adenosine contributes to the renal vasoconstriction observed in the AngII-induced hypertension. This can be either mediated by a decrease in the activity and expression of ADA, increased production of adenosine, or an imbalance in adenosine receptors [111].

Original studies have shown increased platelet aggregation as a result of high ADA activity and expression and lack of adenosine in pregnancy hypertensive [112, 113]. It was indeed suggested that ADA activity as well as platelet aggregation could serve as peripheral markers for the development of therapy for the maintenance of homeostasis and inflammatory processes in hypertension and hypertension-associated pathologies [108, 113]. In contrast, Iriyama et al. have analyzed adenosine metabolism using two different animal models of preeclampsia [114]. They have demonstrated that adenosine levels were high in preeclampsia, and this increase was enough to induce hallmark features of preeclampsia including hypertension, proteinuria, small fetuses, and impaired placental vasculature. This study has also revealed that besides the high levels of adenosine, the receptor A2B was excessively activated, contributing to the development of preeclampsia-related features, although the key finding of this study was that the placental adenosine increase is triggered by the elevation of placental CD73, thereby contributing to preeclampsia [114]. Discrepancies between those two studies can be explained by experimental design differences, for instance, site of analysis (platelets versus placenta). Finally, Iriyama et al. did not evaluate the ADA enzyme. Therefore, more studies are necessary to elucidate whether adenosine causes or protects against preeclampsia and to unveil the actual role of ADA in preeclampsia. This information is summarized in Table 2.

### 3.3. Cancer

Cancers are considered heterogeneous pathologies that vary by tissue of origin and genomic, proteomic, and metabolic alterations [115]. The inflammation plays an important role in the steps required for cancer metastasis because a wide variety of cytokines and other proinflammatory markers contributes to both the extrinsic and intrinsic pathways of inflammation associated by cancer [116].

The enzymatic chain responsible for ATP hydrolysis and adenosine production, present in all immune and vascular cells, has an important role in the control or promotion of inflammation in tumors [117]. This is because of an exuberant immune/inflammatory response, not adequately balanced by endogenous mechanisms of homeostatic control that can lead to persistent and abnormal forms of collateral tissue damage [118]. Adenosine can accumulate in the tumor environment and stroma and generate an immunosuppressed microenvironment that favors the development and metastasis of neoplasias [119].

Multiple mechanisms are involved in adenosine effects, which include inhibition of T helper 1 cell (TH1 cell) cytokine production, deregulation of mononuclear phagocyte cell differentiation and maturation, suppression of effector T cells, and generation of an angiogenic and matrix remodelling environment that is suitable for cancer growth [120, 121]. Most of the signaling actions of extracellular adenosine are mediated by G protein-coupled cell surface receptors that are divided into four subtypes: A1, A2A, A2B, and A3 [122].

The extracellular adenosine acts as a local modulator and exerts effects by protecting cells and tissues from an excessive inflammatory response and as favorable to the onset and cancer growth, by favoring angiogenesis and matrix remodeling [122, 123]. When adenosine active A2A, A2B, and A3 receptors, macrophages promotes the release of the anti-inflammatory cytokine like as TNF, IL-6, IL-10, IL-12, nitric oxide (NO) and macrophage inflamatory protein-(MIP)-1*α* [52, 120].

The A2A receptor activity, a T-cell surface immune checkpoint protein, could lead to the discovery that adenosine in the tumor microenvironment interferes with antitumor immunity, suggesting that antagonism of the A2A could be an effective cancer immunotherapeutic [124].

### 3.4. Epilepsy

Epilepsy is a debilitating neurological disease, characterized by recurring, spontaneous, unprovoked seizures. Temporal lobe epilepsy (TLE) is the most common form of acquired epilepsy, affecting people from all ages [125]. The World Health Organization (WHO) reports that epilepsy affects approximately 50 million people worldwide, with about a third of patients either resistant to current antiepileptic drugs (AED) or experiencing unacceptable side effects.

Neuroinflammation is increasingly recognized as one of the key players in seizure generation and propagation and in the maintenance of the epileptic phenotype [126]. During pathological conditions with increased neuronal firing or cell death nucleotides, ATP is released into the extracellular space in the brain. Although studies show discrepancies about ATP release during seizures and chronic epileptic state, this is possibly due to ATP rapid hydrolysis, by ectonucleotidases in the extracellular space, making it difficult to directly measure its release [127]. Once released, ATP activates P2 receptors (P2X and P2Y), mediating the release of gliotransmitters, which triggers neuronal hyperexcitability and neuroinflammation. Taking advantage of experimental models of epilepsy, the expression and function of P2X receptors have been well established in research field [36, 128]. However, only recently has been suggested that the P2Y receptor subfamily plays a relevant role in experimental and human epilepsy [67, 129]. Furthermore, while adenosine anticonvulsant properties are well established [130], the contribution of extracellular nucleotides to seizures and epilepsy pathology is an area to be further explored [36]. Therefore, not only the targeting of molecules involved in inflammatory pathways but also the targeting of purinergic signaling or their combination seems relevant approaches for developing novel therapies for epilepsy [36, 126].

### 3.5. Alzheimer's Disease

Alzheimer's disease (AD), the leading cause of dementia worldwide, is a progressive neurodegenerative disease associated with the deposition of *β*-amyloid peptide (A*β*) within the brain, along with intracellular neurofibrillary tangles (NFTs) mainly formed by hyperphosphorylated tau protein [131]. The impairment of mitochondrial function and reduction of ATP levels are pathological conditions found in AD, which is closely linked to the decline of cognitive processes [132, 133]. It is well known that in the brain, ATP is secreted by the neurons, glia, and endothelial cells that constitute the blood-brain barrier. Coincident with its release is the secretion of soluble ectonucleotidases which control the effective ATP concentration by breaking it down to adenosine [134]. The involvement of ectonucleotidases on the process of learning and memory in rats has already been described [135–137]. It has also been shown that P2X7 purinergic receptors are upregulated in the brain of patients with AD and in animal models [138, 139]. Interestingly, inhibition of P2X7 in mice, transgenic for mutant human amyloid precursor protein, reduced the number of amyloid plaques in the hippocampus [140] and stimulation of P2X7 receptors on human macrophages and microglia enhanced the degenerative lesions observed in AD [141] (Tables 3 and 4). Furthermore, direct alterations of purine metabolism have been detected in AD by metabolomics, in the ventricular cerebrospinal fluid at postmortem [142], cerebrospinal fluid in living individuals [142–144], and AD brains [145].

Ectonucleotidase activities may be involved in the early events related to memory acquisition and consolidation of an aversively motivated learning task [135, 136]. Previous studies performed in animals subjected to scopolamine model of dementia, which mimics the memory deficit observed in diseases characterized by impairment in cholinergic neurotransmission, such as AD [146–148], have shown a marked reduction in the ATP levels and changes in ectonucleotidase activities (E-NTPDase, E-5′-nucleotidase, and ADA), in the cerebral cortex and hippocampus of rats [149, 150].

Tissue-nonspecific alkaline phosphatase (TNAP) is one of four alkaline phosphatase isozymes that has been described as an ectonucleotidase being able to cleave all forms of adenosine phosphates, influencing purinergic signaling [48]. Preclinical assays tested on more than 100 AD patients have demonstrated that TNAP activity is significantly increased in the hippocampus of AD patients compared with age-related controls [151]. In fact, it has been shown that the extracellular hyperphosphorylated tau protein coming from damaged neurons must be dephosphorylated to become an agonist of a muscarinic receptor [152]. This event has been associated with increased TNAP expression and unbalances of the intracellular calcium homeostasis and phosphorylation levels of intracellular tau [153]. Increased TNAP levels in the plasma of the AD patients have also been reported in literature [151, 154], suggesting that TNAP is a good biomarker of disease progression. On the other hand, the activity of the purinergic enzyme ADA did not present any significant differences in the serum of patients with AD, compared to unaffected controls [155].

### 3.6. Parkinson's Disease

Parkinson's disease (PD) is considered the most frequent movement disorder. Clinically, “movement disorder” is defined by its cardinal motor symptoms: bradykinesia, rigidity, and tremor at rest [156]. Furthermore, most patients will also experience nonmotor symptoms that include mood, cognitive, speech, sensation, and sleep disturbances. The major cause of these symptoms is the progressive loss of dopaminergic neurons in the substantia nigra pars compacta projecting to the striatum, leading to a severe deficiency of dopamine in the putamen and the caudate nucleus. The neuropathological hallmark of PD is cytosolic Lewy bodies, which are characterized by aggregated *α*-synuclein (*α*-Syn) [157].

Activated microglia is a common feature observed in individuals with neurodegenerative disorders, including PD, and it possibly contributes to neuronal death. Jiang et al. have shown that stimulation of the microglial P2X7 receptor by extracellular *α*-Syn, with PI3K/AKT activation and increased oxidative stress, could be an important mechanism and a potential therapeutic target for PD [158]. Furthermore, release of ATP from disrupted cells might cause cell death in neighboring cell-expressing P2X7 receptors, leading to a necrotic volume increase, which has also been implicated in the pathogenesis of PD [159]. Recently, several reports have suggested potential effects of the adenosine A2A receptor antagonist on cognitive dysfunction in PD [160, 161]. Indeed, in rodent models of PD, A2A antagonism exerts antiparkinsonian actions [162–164]. Similarly, this treatment proved to be effective against experimentally induced tremor [165].

Despite several studies provide strong evidence that purinergic receptors are linked to the pathogenesis of PD, the evidence available on a potential involvement of purinergic enzymes is still very scarce. To date, we have only found a few articles concerning this issue. Medeiros et al. have studied the levels of ATP and the activity of the enzymes ADP E-NTPDases, ecto-5′-nucleotidase, and ADA in patients with PD [166]. The results show higher E-NTPDase ATP/ADP activities in PD patients, suggesting an important inflammatory activity in this disease. Another study has analyzed the mRNA levels of E-NTPDase and E-5′-nucleotidase from striatal slices, in a unilaterally lesioned 6-OHDA rat model [167]. The classical method of intracerebral infusion of 6-OHDA involving a massive destruction of nigrostriatal dopaminergic neurons is largely used to investigate motor and biochemical dysfunctions in PD [168]. However, it was found no alteration in the mRNA levels of the enzymes tested [167] (Tables 3 and 4).

### 3.7. Multiple Sclerosis

Multiple sclerosis (MS) is a chronic inflammatory disease, of progressive character with relapsing-remitting phases, that represents the major disabling neurological illness of young adults [176]. This disease affects the myelin sheath of axons and is characterized by demyelination of neurons in the central and peripheral nervous system. Furthermore, the establishment of tissue oxidative stress associated with a neuroinflammatory process culminates in the loss of myelin content and subsequent death of oligodendrocytes and Schwann cells [177, 178].

The mechanisms implicated in the pathogenesis of MS include autoimmunity, inflammation, demyelination, and neurodegeneration. Since the beginning of demyelination processes, the appearance of multiple plates in the white matter of brain and spinal cord can occur [179]. These lesions lead to the disability or complete loss of nerve impulses that result in the appearance of clinical manifestations such as motor impairment [180], blurred vision, hyperalgesia in the upper and lower limbs, excessive tiredness, weakness, anxiety, depression, and irreversible neurological deficits [179, 181, 182]. The remission is a subsequent phase characterized by the remyelination and tissue repair, with partial or complete disappearance of symptoms [176].

The relationship between inflammation and degeneration during the progression of MS is due to successive outbreaks and remissions. There is a higher frequency of events accompanied inflammatory demyelination of the white matter and damage to axons. In this step, it is possible to tissue repair capacity in which there is a remyelination and axonal in the disappearance of clinical manifestations. As these events are repeated, the tissue recovery becomes ineffective. The secondary progressive phase initiates with the establishment of neurodegenerative tissue followed by cerebral gray matter atrophy. At this stage, there is a progressive increase over the score disabilities that can lead to incapacitation of the patient [183].

The MS does not have etiology and cure defined. Therefore, several types of animal models that produce demyelination have been used for scientific research, in order to discover pharmacological tools for assessing compounds as potential drugs or search for evidence of cellular mechanisms to better understand this disease. Among these, it is possible to highlight the chemically induced CNS lesions, such as those generated with cuprizone feeding, ethidium bromide injection, and lysolecithin, or the experimental autoimmune encephalomyelitis (EAE) model, widely used in drug screening as it most faithfully represents the pathology seen in MS [184]. As described by Merrill, the advantages of these models are the dissociation of the demyelination event, from the complexities introduced into the tissue pathology by chronic inflammatory cells and their soluble mediators, reproducibly timed spontaneous remyelination, and robust demyelination and remyelination in anatomically distinct areas facilitating focused, quantitative assessment of lesion generation and repair [185–187].

Recent reports have highlighted the association between receptors and signaling molecules of the purinergic system with demyelinating diseases, including MS [188–191]. Furthermore, ATP and adenosine also play a significant role in the pathophysiology of numerous acute and chronic disorders that include events related with CNS demyelination and remyelination [23, 192–194].

Over the last decade, the ectonucleotidases have become target of study by modulating purinergic signaling and contributing to the fine-tuning of inflammatory and immune responses. The overwhelming evidence indicates that extracellular ATP acting through specific cell surface receptors is involved in proinflammatory functions such as stimulation and proliferation of lymphocytes and microglial cell and cytokine production and secretion [195, 196]. However, its breakdown product, adenosine, exhibits potent anti-inflammatory and immunosuppressive action by inhibiting proliferation of T cells and secretion of cytokines [197, 198].

The association of the purinergic receptors in the demyelinating diseases has been explored in some previously published studies [199, 200]. An overview of the main findings relating the purinoreceptors with MS in experimental models and postmortem tissue of humans with this pathology can be found in Table 5.

Based on the evidence reported in Table 2, the four purinergic receptors especially known to be involved in MS are P2X7, P2Y12, A1, and A2A receptors. Although the role of purinoreceptors is not well elucidated, it is important to note that the number of published evidence and studies for MS is superior to investigations into the role of ectonucleotidases in this pathology. In this way, in Table 2, we also reviewed the activity and expression of ectonucleotidase enzymes ecto-NTPDase, E-5′-nucleotidase, and adenosine deaminase in experimental models and in patients with MS (see Table 6).

## 4. Conclusions

The main causes of death and sickness around the world have changed significantly in the last years, with chronic neurologic and degenerative diseases becoming more and more important. Several evidences show that purinergic signaling system is involved in processes associated with health and disease. In this way, the role of the purinergic receptors in the pathophysiology of different degenerative diseases has been extensively studied. However, the role of the purinergic system enzymes is not fully understood yet. This review summarizes the most current knowledge on the role of families of nucleotide metabolizing enzymes, the ectonucleotidases, on degenerative diseases. Considerable attention was directed towards diseases related to the cardiovascular system (atherosclerosis and hypertension) and central nervous system (epilepsy, Alzheimer's disease, Parkinson's disease, and multiple sclerosis). Firstly, it has been shown that a reduction in the activity of ectonucleotidases can be associated with progression of arteriosclerosis. Furthermore, high expression and activity of CD73 and E-NTPDase 1 may have beneficial effects contributing to the production of extracellular adenosine. In parallel, the low expression and activity of ADA are an alternative maintenance of levels of adenosine, in order to increase antithrombotic and anti-inflammatory effects. In relation to hypertension, a suppression of E-NTPDase 1 is linked to the pathogenesis of this disease and an increase in the activity of ectonucleotidases was reported in animal models of hypertension and in human. On the other hand, P2 receptor modulation seems to be a promising tool for a novel therapeutic approach for epilepsy. Lastly, a reduction of ATP levels in experimental models for AD and increase in the ectonucleotidases activities, especially E-NTPDase in patients with Parkinson's disease and multiple sclerosis, were found.

In conclusion, we verified that the activity and expression of the ectonucleotidases investigated in this review may be altered in some degenerative diseases. Taken together, the data discussed in this paper collaborate for a better understanding of the molecular mechanisms involved in these highly complex diseases, suggesting a number of directions for future research. In fact, more studies should be conducted to better understand the changes in metabolism and maintenance of adenine nucleotide and nucleoside levels in degenerative diseases. In addition, the study of the ectonucleotidase role may contribute to a better understanding of the molecular mechanisms of highly complex diseases and with major impact on patients' quality of life.

## 5. Future Prospects

Ectonucleotidases are nucleotide-metabolizing enzymes and play an essential role in regulating and controlling extracellular nucleotides. These nucleotides and nucleosides are responsible for the well functioning of normal cells. A wrong signaling mediated by these molecules triggers pathophysiological disorders like as atherosclerosis, hypertension, cancer, epilepsy, Alzheimer's disease, Parkinson's disease and multiple sclerosis. In this way, understanding the role of these molecules can be an important key factor to discover pharmacological targets and mechanisms that minimize deleterious effects of these diseases.

Until now, the investigations describes which ectonucleotidases have their activity or expression changes in different biological tissues in experimental models and patients with the pathologies reviewed in this study. From these findings, the next step is to know how the catabolic activity of ectonucleotidases can regulate the production of proinflammatory mediators and the immune response, both physiologically and pathologically.

Evidences have pointed out the important role of inhibitors of ectonucleotidases, highlighting their potential as novel drugs [220–222]. It is suggested that inhibitors of ectonucleotidases might be an ideal drug target for many therapeutic applications such as anticancer and immunomodulatory for the treatment of central nervous system disorders and cardiovascular diseases [220]. An important perspective would be to explore the use of these drugs in experimental biology. Such investigations would reveal whether up- or downregulation of the ectonucleotidases would restore immune markers and physiological functions compromised in experimental models and cell cultures. Since inhibitors of ectonucleotidases might be an ideal drug target for many therapeutic applications such as anticancer and immunomodulatory for the treatment of central nervous system disorders and cardiovascular diseases [220], more research is needed to elucidate the role of these enzymes in these pathologies and their role potential targets for drug discovery.

## Figures and Tables

**Table 1:** Ectonucleotidases (E-NTPDase, E-5′-nucleotidase, and ADA) and purinergic receptors in experimental models and patients with atherosclerosis.

Sample	E-NTPDase	E-5′-nucleotidase	Adenosine deaminase	Involved receptors	Reference
Endothelial cells and thoracic aorta from mouse model of atherosclerosis	↓ enzymatic activity and expression	—	—	—	[72, 84]

ApoE-deficient mice	—	↓ enzymatic activity and expression	—	—	[88]

Platelets and plasma from patients	↓ enzymatic activity	↑ enzymatic activity	—	—	[90]

Plasma of experimental model	—	—	↑ enzymatic activity	A1 upregulation	[93]

Atherosclerotic vessel wall from aortoiliac bifurcation of Apoliprotein E- and LDL-deficient animals	—	—	↓ enzymatic activity	—	[92]

**Table 2:** Ectonucleotidases (E-NTPDase, E-5′-nucleotidase, and ADA) and purinergic receptors in experimental models and patients with hypertension.

Sample	E-NTPDase	E-5′-nucleotidase	Adenosine deaminase	Involved receptors	Reference
Treg cells from angiotensin II-dependent hypertension	↑ expression CD39 ↑ enzymatic activity to ATP and ADP	—	—	—	[99]

Pulmonary arterial hypertension	↑ expression CD39 ↑ enzymatic activity to ATP and ADP	—	—	—	[100, 101]

Preeclampsia	↑ expression CD39 can protect against preeclampsia	—	—	—	[102]

Synaptosomes from rat heart	↑ expression of NTPDase 2	↑ expression			[77, 103]

Human coronary arteries	—	↑ expression	—	P1 upregulation	[104]

Platelets and lymphocytes of hypertensive human	↑ expression ↑ enzymatic activity	↑ enzymatic activity	↑ enzymatic activity	—	[109–111]

Animal membrane fractions of renal tissue	—	—	↓ enzymatic activity and expression	A2A downregulation A3 upregulation	[113]

Platelet of pregnant hypertensive woman	—	—	↑ enzymatic activity	—	[114, 115]

Placenta of pregnant hypertensive woman	—	↑ expression ↑ enzymatic activity	—	—	[116]

**Table 3:** Role of purinoreceptors in experimental models and patients with Alzheimer's disease (AD) and Parkinson's disease (PD).

R Sample	P2X7 receptor	A2A receptor	Reference
Transgenic mouse model of AD (brain slices)	Upregulated	—	[169]

Microglia from AD patients Human microglia treated with amyloid-beta peptide *in vitro* Rat hippocampus after amyloid-beta peptide injection	Upregulated	—	[170]

Transgenic mouse model of AD	Inhibition of P2X7R decreased the number of hippocampal amyloid plaques	—	[171]

Human macrophages and microglia preactivated with amyloid-beta peptide	P2X7R stimulation enhanced secretion of proinflammatory cytokines	—	[172, 173]

Rats injected with 6-OHDA	—	Inhibition of A2AR improved motor performance and cognition	[174]

Rats injected with haloperidol (DA antagonist)	—	Inhibition of A2AR reversed locomotor suppression and tremulous jaw movements	[174, 175]

Alzheimer's disease (AD), P2X7 receptors (P2X7R), adenosine A2A receptor (A2AR), 6-hydroxydopamine (6-OHDA), and dopamine (DA).

**Table 4:** Ectonucleotidases (E-NTPDase, E-5′-nucleotidase, and adenosine deaminase) in experimental models and patients with Alzheimer's disease (AD) and Parkinson's disease (PD).

R Sample	E-NTPDase	E-5′-nucleotidase	Adenosine deaminase	Alkaline phosphatase (TNAP)	Reference
Synaptosomes of rats injected with scopolamine	↓ enzymatic activity to ATP (hippocampus and cortex) ↓ enzymatic activity to ADP (hippocampus)	↓ enzymatic activity (hippocampus)	↓ enzymatic activity (hippocampus)	—	[169]

Synaptosomes of rats injected with scopolamine	↑ enzymatic activity to ATP (hippocampus and cortex) ↑ enzymatic activity to ADP (hippocampus)	↓ enzymatic activity (hippocampus)	↑ enzymatic activity (hippocampus)	—	[170]

Hippocampus and plasma from patients with AD	—	—	—	↑ enzymatic activity	[171]

Plasma from patients with AD	—	—	—	↑ enzymatic activity	[172, 173]

Serum from patients with PD	↑ enzymatic activity to ATP ↑ enzymatic activity to ADP	—	—	—	[174]

Alzheimer's disease (AD) and Parkinson's disease (PD).

**Table 5:** Role of purinoreceptors in experimental models and patients with MS: overview of main findings.

Sample	Receptors	Main findings	Reference
Cerebral cortex from healthy and MS patients	P2Y12 receptor	Reduction in the P2Y12R is immunoreactive in the lesions. This event was directly correlated with the extent demyelination found in grey matter cortical and subcortical white matter.	[201]

Tissues of rats exposed to EAE model and brain tissue from healthy and MS patients	P2X7 receptor P2Y12 receptor	P2X7R is highly expressed in microglia in MS lesions during the peak of EAE. P2X7R is associated with a proinflammatory phenotype of human microglia. In parallel, P2Y12R was associated with an anti-inflammatory phenotype in human microglia. P2Y12R was expressed at lower levels in active inflammatory MS lesions. P2Y12R expression increased in the remission phase of EAE.	[202]

Spleen and lymph node cell from P2X7R^−/−^ mice exposed to EAE model	P2X7 receptor	Coculture of P2X7R^−/−^ macrophages with wild-type lymphocytes showed that enhanced proliferative activity resided within the P2X7R^−/−^ lymphocyte population. Furthermore, mRNA and protein for IFN-*γ* were significantly reduced in the CNS of P2X7R^−/−^ mice with EAE. Enhanced susceptibility of P2X7R^−/−^ mice to EAE reflects a loss of apoptotic activity in lymphocytes.	[203]

EAE induced in rats by guinea pig spinal cord homogenates (GPSCH model)	A1 receptor	Investigation of the role of the A1 receptor using antagonists. Caffeine (10–30 m/kg) decreases the incidence of EAE and attenuates EAE pathology at behavioral, histological (inflammatory cell infiltration and demyelination), and neurochemical (expression of inflammatory cytokines) levels. In addition, caffeine also upregulated A1 receptor and TGF-*β* mRNAs and suppressed INF-*γ* mRNA in EAE rats.	[204]

Brain of female Lewis rats exposed to EAE model	P2X7 receptor	Enhanced expression of GFAP and S100*β* is associated with expression of P2X7R. Brilliant blue G, an antagonist of P2X7R, significantly decreases astrogliosis (GFAP and S100*β*).	[205]

Brain of rats exposed to EAE model	P2X7 receptor	The enhancement in the expression of the P2X7 receptor at the level of both mRNA and protein was observed in the peak of neurological symptoms and was connected mostly with neurons (4, 6, 8, and 10 days postimmunization).	[206]

Human monocytes from Australasian patients with MS	P2X7 receptor	A rare P2X7 variant Arg307Gln with absent pore formation function protects against neuroinflammation in MS.	[207]

Two independent mouse EAE models: by immunization in C57BL/6 using MOG_35–55_ and by PLP_139–155_ in mouse wild-type and lacking A2AR (A2AR^−/−^)	A2A receptor	Upregulation of A2AR in the CNS in EAE, predominantly detected on T cells and macrophages/microglia. A preventive EAE treatment with A2AR-specific agonist inhibited myelin-specific T cell proliferation ex vivo and ameliorated disease. In parallel, the application of the same agonist after disease onset exacerbated nonremitting EAE progression and resulted in more severe tissue destruction. A2AR-deficient mice showed accelerated and exacerbated disease manifestation with higher numbers of inflammatory lesions in the early stage. EAE quickly ameliorated and myelin debris accumulation was lower in A2AR^−/−^ mice. Finally, an in vitro activation of A2AR inhibited phagocytosis of myelin by macrophages and primary microglia as well as migration of CD4^+^ T cells, macrophages, and primary microglia.	[194]

Lymphocyte isolation from nerve tissue and lumbar spinal cord of female mice exposed to EAE	A2A receptor	CGS21680 (CGS, A2AR agonist) significantly suppressed specific lymphocyte proliferation, reduced infiltration of CD4^+^ T lymphocytes, and attenuated the expression of inflammatory cytokines, which in turn inhibited the EAE progression. CGS can increase the [Ca2^+^]i in murine lymphocytes, which may be the mechanism underlying the suppressive effects of CGS-induced A2AR activation on EAE progression.	[208]

Oligodendrocyte cultures and postmortem optic nerve samples from MS patients	P2X7 receptor	Sustained activation of P2X7R in vivo causes lesions that are reminiscent of the major features of MS plaques (demyelination, oligodendrocyte death, and axonal damage). In addition, treatment with P2X7R antagonists reduces demyelination and ameliorates the associated neurological symptoms. The study suggests that ATP can kill oligodendrocytes via P2X7R activation and this process contributes to EAE. Importantly, P2X7R expression is elevated in normal-appearing axon tracts in MS patients.	[209]

Peripheral blood mononuclear cells from MS patients	A1 receptor	Decreased levels of adenosine and its A1 receptor modulate TNF*α* and IL-6 levels and may contribute to the pathogenesis of MS.	[210]

Brain and spinal cord of female SJL/J mice infected with Theiler's virus infection	A2A receptor	A2A receptors participate in anti-inflammatory effects of cannabidiol. A2A antagonist ZM241385 partially blocks the protective effects of cannabidiol in the initial stages of inflammation.	[211]

Human microglia	P2Y12 receptor	P2Y12 is expressed on parenchymal microglia and is stable throughout human brain development, including fetal phases. MS result in decreased P2Y12 immunoreactivity in plaque- or lesion-associated myeloid cells. P2Y12 is a useful marker for the identification of human microglia throughout the lifespan.	[212]

Blood from MS patients	P2X4 receptor P2X7 receptor	A rare genetic variant in P2RX4 and P2RX7 is a major genetic contributor to disease (description of the three variant haplotypes: P2RX7 rs140915863:C>T [p.T205M]; P2RX7 rs201921967:A>G [p.N361S]; and P2RX4 rs765866317:G>A [p.G135S]).	[213]

C57BL6 mice and P2X7-deficient mice exposed to EAE model	P2X7 receptor	The incidence of EAE disease in P2X7 mice was reduced 4-fold compared to the wild type. Mouse splenic T cells isolated from P2X7 null mice produced greater IFN*γ* and IL-17 (from 3- to 12-fold greater levels) than wild-type cells. Although infiltrating cells were detected in the brains of both the P2X7 and wild type, astroglial activation and axonal damage were reduced compared to wild type.	[214]

Brain and spinal cord from female mice exposed to EAE model	A1A receptor	A1AR^−/−^ mice developed a severe progressive-relapsing form of EAE compared with their wild type. Demyelination, axonal injury, and enhanced activation of microglia and macrophages were observed in A1AR^−^/^−^. Spinal cords from A1AR^−/−^ mice demonstrated increased proinflammatory gene expression. A1AR^−/−^ macrophage-derived soluble factors caused significant oligodendrocyte cytotoxicity compared with wild-type controls.	[215]

Spinal cord from MS patients	P2X7 receptor	In control spinal cord, few small microglial cells/macrophages were scattered throughout the tissue. However, MS specimens had significantly greater density of such cells with longer processes in affected regions. MS also had significantly greater density of P2X7 and immunoreactive microglial cells/macrophages in affected regions.	[216]

MS (multiple sclerosis), experimental autoimmune encephalomyelitis (EAE), central nervous system (CNS), and glial fibrillary acid protein (GFAP).

**Table 6:** Ectonucleotidases (E-NTPDase, E-5′-nucleotidase, and ADA) in experimental models and patients with MS: a review.

Sample	E-NTPDase	E-5′-nucleotidase	Adenosine deaminase	Reference
Serum of MS patients (RRMS form)	—	—	↑ enzymatic activity	[169]

Lymphocytes of MS patients (RRMS form)	↑ expression CD39 ↑ enzymatic activity to ATP and ADP	—	↓ enzymatic activity	[170]

Platelets of MS patients (RRMS form)	↓ enzymatic activity to ATP and ADP	↓ enzymatic activity	↓ enzymatic activity	[171]

Platelets from rats demyelinated with EB	↓ enzymatic activity to ATP (on days 3, 7, 15, and 21) ↓ enzymatic activity to ADP (on day 7)	↓ enzymatic activity (on day 15)	—	[172, 173]

Platelets from rats demyelinated with EB	↑ enzymatic activity to ATP (on day 7) ↑ enzymatic activity to ADP (on day 7)	↑ enzymatic activity (on day7)	—	[174]

Synaptosomes from cerebral cortex of rats demyelinated with EB	↑ enzymatic activity to ATP (on days 7 and 15) ↑ enzymatic activity to ADP (on day 7)	↑ enzymatic activity (on day 7)	—	[174, 175]

Plasma membrane from lumbosacral region of spinal cords from EAE-induced rats	↑ enzymatic activity to ATP (on days 7, 15, and 25) ↑ enzymatic activity to ADP (on day 15)	↑ enzymatic activity (on days 15 and 25)	—	[217]

Blood serum from EAE-induced rats	↑ enzymatic activity to ATP (on day 25) ↓ enzymatic activity to ADP (on days 15 and 25)	↓ enzymatic activity (on day 15)	—	[217]

T cells from patients with relapsing-remitting MS	↓ in number of CD39-positive Treg cells	—	—	[218, 219]

EB (ethidium bromide), relapsing-remitting multiple sclerosis (RRMS), experimental autoimmune encephalomyelitis (EAE), and Treg (regulatory T cells).
